# Mitochondrial apoptosis and BH3 mimetics

**DOI:** 10.12688/f1000research.9629.1

**Published:** 2016-12-01

**Authors:** Haiming Dai, X. Wei Meng, Scott H. Kaufmann

**Affiliations:** 1 Division of Oncology Research , Mayo Clinic, Rochester, MN, 55905, USA; 2 Department of Molecular Pharmacology & Experimental Therapeutics, Mayo Clinic, Rochester, MN, 55905, USA; 3 Center for Medical Physics and Technology, Hefei Institute of Physical Science, Chinese Academy of Sciences, Hefei, 230031, China

**Keywords:** BCL-2 inhibitors, venetoclax, BAX activation, BAK activation, BH3 mimetics

## Abstract

The BCL2-selective BH3 mimetic venetoclax was recently approved for the treatment of relapsed, chromosome 17p-deleted chronic lymphocytic leukemia (CLL) and is undergoing extensive testing, alone and in combination, in lymphomas, acute leukemias, and solid tumors. Here we summarize recent advances in understanding of the biology of BCL2 family members that shed light on the action of BH3 mimetics, review preclinical and clinical studies leading to the regulatory approval of venetoclax, and discuss future investigation of this new class of antineoplastic agent.

## Introduction

The recent regulatory approval of venetoclax for the treatment of chronic lymphocytic leukemia (CLL) culminates 30 years of investigation in many labs worldwide. Milestones in this effort have included the cloning of *BCL2* at the t(14;18) translocation in follicular lymphomas ^1, 2^, demonstration that BCL2 inhibits cell death ^3, 4^, realization that BCL2 is elevated in CLL ^5, 6^, recognition that BCL2 and its anti-apoptotic paralogs bind BH3-only proteins through their BH3-binding grooves ^7^, identification of ABT-737 and navitoclax as BH3-binding groove-directed inhibitors of BCL2 and BCLX _L_^8, 9^, demonstration that navitoclax is active against CLL ^10^, and derivation of venetoclax as a BCL2-selective BH3 mimetic ^11^. While the approval of venetoclax for CLL is a triumph in its own right, the challenge remains to optimize the use of this agent and other BH3 mimetics for improved therapy of diverse malignancies. To provide context for these ongoing efforts, we review recent progress in understanding the action of BCL2 family proteins, summarize the clinical status of venetoclax and other BH3 mimetics, and discuss possible approaches to predicting whether various cancers will respond to these agents.

## Mitochondrial apoptosis and BAX/BAK activation

BH3 mimetics are designed to inhibit anti-apoptotic BCL2 family proteins, leading to BAX and BAK activation ^12– 14^. Accordingly, recent advances in understanding the functions of various BCL2 family members provide important insight into the therapeutic effects of BH3 mimetics.

### Mitochondrial apoptosis

BCL2 family members regulate apoptosis, a distinct form of cell death that plays critical roles in development, immune response, and tissue homeostasis ^15– 17^. This type of cell death can be triggered through two different pathways depending on the stimulus. The death receptor pathway is initiated through binding of death ligands to certain cell surface receptors. In contrast, the mitochondrial or intrinsic apoptotic pathway involves the release of mitochondrial intermembrane proteins, including cytochrome c and Smac/Diablo, to the cytosol, where they contribute to subsequent apoptotic changes ^18– 20^. The translocation of these intermembrane proteins is modulated by the BCL2 family of proteins.

Based on differences in structure and function, BCL2 family members are divided into three subgroups ^20– 22^: BAX and BAK, which contain three distinct BCL2 homology (BH) domains and, upon activation, permeabilize the mitochondrial outer membrane (MOM) by forming proteinaceous pores ^23– 26^ or in other ways ^27– 30^; the anti-apoptotic family members BCL2, BCLX _L_, MCL1, BCLW, and BCL2A1 (also called BFL1 in humans and A1 in mice), which typically contain four BH domains and oppose MOM permeabilization; and the BH3-only proteins BIM, BID, PUMA, NOXA, BAD, BIK, BMF, and HRK, which share homology with other BCL2 family members only in their 15-amino-acid α-helical BH3 domain and induce apoptosis by facilitating BAX and/or BAK activation ^22^.

### BAX/BAK activation models

Three different models have been proposed to explain BAX and BAK activation. The direct activation model proposes that certain BH3-only proteins directly interact with BAX and/or BAK to cause a conformational change that leads to BAX/BAK oligomerization and activation ^31– 33^. In this model, the major role of anti-apoptotic BCL2 family members is to inhibit the BH3-only proteins. The indirect activation model proposes that BAX and BAK are tonically activated but are restrained by anti-apoptotic BCL2 family members ^34^. In this model, BH3-only proteins induced by various death signals primarily inhibit the anti-apoptotic BCL2 family members, leading to the release of activated BAX and BAK. Finally, the unified model proposes that anti-apoptotic BCL2 family proteins inhibit both BH3-only proteins and activated BAX or BAK ^35^. In both instances, the exposed BH3 domains of the pro-apoptotic proteins are neutralized by interaction with BH3-binding grooves, extended clefts on the surfaces of anti-apoptotic BCL2 family members ^36, 37^. The BH3 mimetics described below have been identified and developed based on their ability to occupy the same BH3-binding grooves.

### Two mechanisms of BH3 mimetic-induced killing

**Figure 1.**
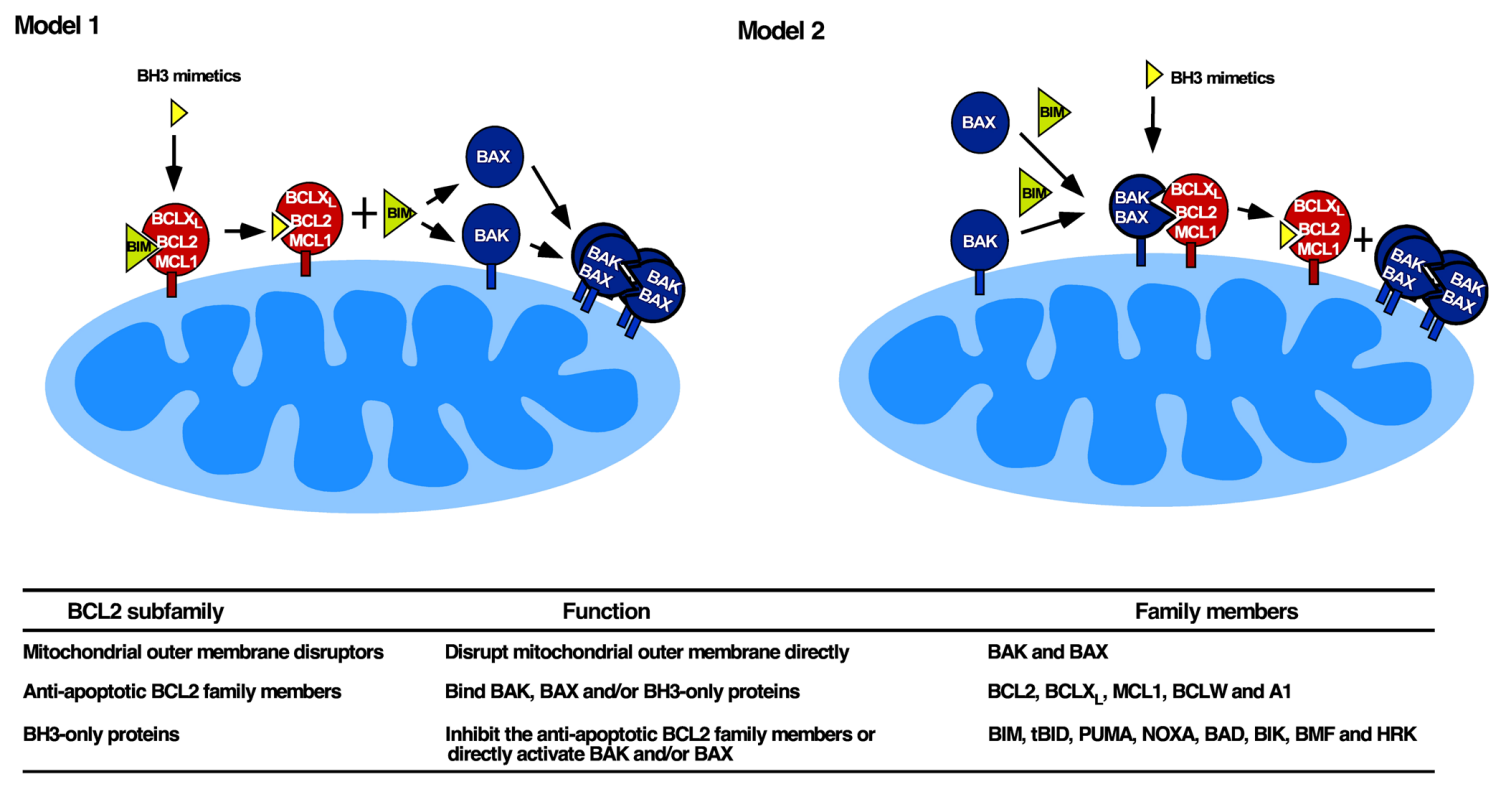
Two models of BH3 mimetic action. In Model 1 (left), BH3 mimetics are thought to displace activated BIM from anti-apoptotic BCL2 family members, allowing BIM to subsequently activate BAX and BAK ^44^. In Model 2 (right), BAK and/or BAX are constitutively activated and are displaced from anti-apoptotic BCL2 family members by BH3 mimetics ^46^. Model 2 is more compatible with recent studies showing that BAK and BAX can be activated in the absence of BH3-only proteins under cell-free conditions ^46^ and in gene-targeted HCT116 cells ^123^. It is, however, possible that one mode of activation predominates in some cell lines or tissues and the other mode predominates in others. **Table below figure,** summary of BCL2 subfamilies, their functions, and members.

Neutralization of BH3-binding grooves on anti-apoptotic BCL2 family members is not, by itself, sufficient to kill cells. Instead, binding of BH3 mimetics to anti-apoptotic BCL2 family members must result in BAX and/or BAK activation to elicit cell death. This BAX/BAK activation can occur by one of two processes ( Figure 1).

First, a subset of BH3-only proteins, termed direct activators, can directly activate BAX and BAK. This group of proteins includes BIM, tBID (a cleaved form of BID), and PUMA ^31, 32, 38^. The role of NOXA as a direct activator has been controversial, with some studies showing activation of BAX or BAK by NOXA protein ^33, 39^ and other studies reporting that NOXA BH3 peptide cannot directly activate BAX or BAK ^40– 42^. Chen *et al.* recently reported that interruption of the gene encoding NOXA in cells already lacking BID, BIM, and PUMA causes increased resistance to multiple apoptotic stimuli, suggesting an important role for NOXA in BAX/BAK activation ^43^. To the extent that BH3-only proteins are constitutively activated but sequestered by anti-apoptotic BCL2 family members ^44, 45^, displacement of BH3-only proteins by BH3 mimetics can provide a driving force for BAX and BAK activation ( Figure 1, Model 1).

Recent results, however, suggest that an alternative mechanism might also contribute to BH3 mimetic-induced killing. In particular, Chen and coworkers reported that BID/BIM/PUMA/NOXA-deficient cells can still undergo apoptosis after certain treatments such as etoposide or ultraviolet light, suggesting the existence of additional BAX/BAK activation pathways ^43^. We simultaneously reported that BAK can undergo lipid-dependent autoactivation under cell-free conditions ^46^. Within intact cells, the extent of constitutive BAK oligomerization (indicative of partial activation) correlated with BAK protein levels across a panel of lymphohematopoietic cell lines. Moreover, BAK knockdown diminished the extent of BAK oligomerization, suggesting concentration-dependent autoactivation *in situ* ^46^.

If BAK undergoes concentration-dependent autoactivation, how can cells with high BAK levels survive? Our further studies demonstrated that constitutively activated BAK is bound to BCLX _L_, MCL1, or less commonly BCL2 ^46^. Based on these observations, BH3 mimetics might be killing cells by displacing partially activated BAK from anti-apoptotic BCL2 family members ( Figure 1, Model 2). Consistent with this possibility, cells with constitutive BAK·MCL1 complexes were particularly sensitive to the MCL1 antagonist A-1210477, whereas those with constitutive BAK·BCLX _L_ complexes were more sensitive to the BCL2/BCLX _L_ inhibitor navitoclax ^46^. In agreement with these observations, mice harboring a BAK mutant with reduced affinity for BCLX _L_ had diminished T cells and platelets, suggesting that BCLX _L_-mediated neutralization of constitutively activated BAK is also important for the survival of certain cell lineages *in vivo* ^47^. In other cells, e.g. human embryonic stem cells, BAX is constitutively activated, likewise conferring sensitivity to BH3 mimetics ^48^.

## Current status of BH3 mimetics

As these complex and highly dynamic interactions between various BCL2 family members were being elucidated, small molecules that could mimic the effects of BH3-only proteins were also being developed. ABT-737, the first compound identified as a bona fide BH3 mimetic ^8^, exhibited high affinity for BCL2, BCLX _L_, and BCLW (K _d_ <1 nM) and lower affinity for MCL1 and A1/BFL1 (K _d_ >1000 nM). Although ABT-737 killed cells in a BAX/BAK-dependent manner ^49, 50^ and exhibited anti-tumor activity ^8^, it was not orally bioavailable and displayed poor aqueous solubility, precluding its clinical development.

### Navitoclax: inhibitor of BCL2, BCLX _L_, and BCLW

Navitoclax (ABT-263), an orally bioavailable small molecule with a binding profile similar to that of ABT-737 ^9^, also disrupts interactions involving BCL2 and BCLX _L_, causes BAX/BAK-dependent apoptosis *in vitro*, and induces complete regressions in xenograft models of small-cell lung cancer (SCLC) and acute lymphoblastic leukemia (ALL) ^9^. In early clinical testing, navitoclax displayed single-agent activity against relapsed/refractory lymphoid malignancies ^10, 51^, especially CLL. Adding navitoclax to the monoclonal anti-CD20 antibody rituximab improved both response rate and progression-free survival in previously untreated CLL compared to rituximab alone ^52^. This hypersensitivity of CLL was thought to reflect frequent deletion of genes on chromosome 13q14 encoding miR15A and miR16A, two microRNAs that normally inhibit BCL2 expression ^53, 54^. Loss of these microRNAs is thought to result in constitutive BCL2 overexpression and BCL2 addiction ^5, 6, 55^.

Unfortunately, navitoclax also acutely induced thrombocytopenia ^10, 51^, reflecting the role of BCLX _L_ in platelet survival ^47, 56, 57^. Although this thrombocytopenia could be diminished by treating patients with 150 mg navitoclax/day for one week followed by therapeutic does of 325 mg daily ^10^, maximal BCL2 inhibition was never achieved in lymphoid malignancies because of toxicities of BCLX _L_ inhibition in other normal tissues. Moreover, clinical activity of navitoclax in solid tumors was limited. SCLC appeared to respond better than did other tumors ^58^, but only 3% (one in 39) of patients ^59^ achieved even a partial response (PR). In addition, when navitoclax was combined with other agents, including carboplatin/paclitaxel ^60^, gemcitabine ^61^, or irinotecan ^62^, extensive toxicity and limited efficacy were observed.

### Venetoclax: a BCL2-selective inhibitor for CLL and beyond

Developed specifically to avoid the thrombocytopenia associated with BCLX _L_ inhibition, venetoclax exhibits selectivity for BCL2 over BCLX _L_ (K _d_ <0.01 nM versus 48 nM, respectively), kills cells in a BAX/BAK-dependent manner, and spares platelets ^11^. In light of the navitoclax clinical results, the first phase I trial of venetoclax was conducted in relapsed or refractory (R/R) CLL, including CLL with deletions of the short arm of chromosome 17 (17p), where the tumor suppressor gene *TP53* is located, unmutated *IGHV*, ** or fludarabine-resistant ** disease ^63^. Among 116 patients treated, 59% achieved PR and 20% clinical complete remission (CR), including 5% who had no detectable residual disease by flow cytometry. A subsequent single-arm phase II trial demonstrated a 72% PR and 7.5% CR rate in R/R CLL with 17p deletion ^64^. The major side effect in these trials was tumor lysis syndrome (TLS), which could be minimized by starting at a dose of 20 mg daily and ramping up weekly to 400 mg daily over 5 weeks. These observations led to FDA approval of venetoclax for 17p-deleted CLL in April 2016 ( http://www.fda.gov/Drugs/InformationOnDrugs/ApprovedDrugs/ucm495351.htm). However, consistent with the idea that venetoclax, as a BH3 mimetic, should induce apoptosis in a *TP53*-independent manner, venetoclax kills CLL cells *ex vivo* regardless of their *TP53* mutation status ^65^. Accordingly, a retrospective analysis of *TP53* status in cases treated in the original phase I trial ^63^ might clarify whether *TP53* wild-type CLL also responds clinically, which could broaden the indication for venetoclax.

Beyond CLL, venetoclax exhibits activity against a variety of lymphoid malignancies. In preclinical studies, concurrent inhibition of BCLX _L_ is required for venetoclax to kill most ALL cells ^66^, the notable exception being MLL-rearranged (MLLr) ALL. In this latter disease, BCL2 is highly expressed because of DOT1L-mediated H3K79 methylation ^67^, rendering MLLr ALL sensitive to venetoclax alone ^66, 67^. A clinical trial of venetoclax in MLLr ALL is awaited with interest.

Venetoclax is also active against lymphomas. In a phase I trial, venetoclax monotherapy had an overall response rate of 44% ( Table 1) in various R/R non-Hodgkin lymphomas ^68^. Addition of the alkylating agent bendamustine and anti-CD20 antibody rituximab resulted in an even more impressive overall response rate in follicular lymphoma, diffuse large B-cell lymphoma, and marginal zone lymphoma ( Table 2) ^69^. In combination with the Bruton’s tyrosine kinase inhibitor ibrutinib, venetoclax also induced remissions in R/R mantle cell lymphoma ^70^.

**Table 1.** Efficacy of venetoclax monotherapy in relapsed/refractory NHL ^a^.

Disease	Number	OR ^b^	CR	PR	Stable	PROG	Median progression- free survival (months)	12-month survival
WM	4	100%	0%	100%	0%	0%	NR	NR
MCL	28	75%	21%	54%	18%	4%	14	82%
MZL	3	67%	0%	67%	0%	0%	NR	NR
DLBCL-RT	7	43%	0%	43%	29%	14%	NR	NR
DLBCL	34	18%	12%	6%	24%	56%	1	34%
FL	29	38%	14%	24%	59%	4%	11	100%
Total	106	44%	13%	31%	30%	22%	17	72%

^a^Summarized from 68. ^b^Abbreviations: CR, complete remission; DLBCL, diffuse large B-cell lymphoma; FL, follicular lymphoma; MCL, mantle cell lymphoma; MZL, marginal zone lymphoma; NHL, non-Hodgkin lymphoma; NR, not reported; OR, overall response rate; PR, partial remission, PROG, progressive disease; RT, Richter’s transformation; WM, Waldenstrom’s macroglobulinemia.

**Table 2.** Efficacy of bendamustine/rituximab/venetoclax against NHL ^a^.

	FL ^b^	DLBCL	MZL	
Number of patients	27	16	6	
OR	78%	38%	80%	
CR	30%	25%	20%	
PR	48%	13%	60%	
Stable	4%	13%	0%	
PROG	7%	38%	0%	

^a^Summarized from 67. ^b^Abbreviations: DLBCL, diffuse large B-cell lymphoma; CR, complete remission; FL, follicular lymphoma; MZL, marginal zone lymphoma; NHL, non-Hodgkin lymphoma; OR, overall response; PR, partial remission, PROG, progressive disease.

BCL2 has also been implicated in the survival of multiple myeloma (MM) cells, particularly those with t(11;14) translocation. Accordingly, MMs with this translocation have a higher response rate to venetoclax than those without (24% versus 4%) ^71^. In addition, a trial of venetoclax in combination with bortezomib and dexamethasone in R/R MM appears promising ^72^.

Venetoclax has also been extensively studied in acute myelogenous leukemia (AML). A preclinical study suggested that AML is exquisitely sensitive to single-agent venetoclax *ex vivo* ^73^. A subsequent phase II clinical trial, however, demonstrated responses (CR/CRi) in only six of 32 patients (19%) with R/R AML ^74^. This somewhat low response rate may be related to the upregulation of BCLX _L_ and MCL1 in this disease, particularly at the time of AML relapse ^75^, as well as other factors such as *HOX* gene expression ^76^. Interestingly, combinations of venetoclax with low-dose cytarabine or DNA methyltransferase inhibitors exhibit response rates of 44% ^77^ and 76% ^78^, respectively, in elderly patients with previously untreated AML, raising the possibility that using venetoclax as a sensitizing agent might be particularly effective in this patient population.

In most solid tumors, BCLX _L_ and MCL1 appear to be more important than BCL2 in inhibiting apoptosis ^79^. However, SCLC ^80^ and estrogen receptor-positive (ER ^+^) breast cancer ^81^ exhibit high BCL2 expression and venetoclax sensitivity. Accordingly, clinical studies of venetoclax in these malignancies appear to be warranted but have not yet been initiated.

### BCLX _L_ inhibitor: WEHI-539

BCLX _L_ is frequently expressed at high levels in solid tumors, including colorectal ^82^, hormone-refractory prostate ^83^, and mesenchymal breast cancers ^84^, conferring chemotherapy resistance ^84, 85^. A BCLX _L_ inhibitor could be particularly useful in treating these cancers. WEHI-539 ^86^ and its more potent derivative A-1155463 ^87^ selectively and tightly bind to BCLX _L_. In mice, A-1155463 causes reversible on-target thrombocytopenia and inhibits SCLC xenograft growth ^87^. In addition, WEHI-539 synergizes with carboplatin in ovarian cancer cell lines ^88^ and with doxorubicin in osteosarcoma ^89^.

Because of the role of BCLX _L_ in platelet survival ^47, 56, 57^, clinical application of a selective BCLX _L_ inhibitor could be a challenge. As with navitoclax, a possible lead-in dose of BCLX _L_ inhibitor may lower the risk of severe thrombocytopenia, but clinical efficacy might also be compromised because suboptimal BCLX _L_ inhibition during the lead-in might facilitate the development of tumor cell tolerance. Therefore, development of an alternative strategy to mitigate thrombocytopenia might be important for the successful application of BCLX _L_ inhibitors.

### MCL1 inhibitor: A-1210477

MCL1 is also an attractive target. MCL1 elevation occurs in many tumors ^75, 79, 90, 91^ and is associated with poor prognosis ^92– 98^. Moreover, MCL1 contributes to therapy resistance ^99^, especially to ABT-737, navitoclax, and venetoclax ^49, 100– 103^.

To date, only one MCL1-selective inhibitor, A1210477, has been reported ^102^. This agent disrupts complexes of MCL1 with BH3-only proteins, kills MCL1-dependent cells, and exhibits synergy with navitoclax *in vitro* ^102, 104^. Studying an agent with these properties *in vivo* could potentially be productive.

## Prediction of BH3 mimetic sensitivity or resistance

Even though venetoclax has substantial activity against 17p-deleted CLL, responses are not universal. In an era of increasing emphasis on precision medicine, there is substantial interest in predicting which cases will respond to venetoclax or other BH3 mimetics and which will not.

### BCL2 family protein levels

Several groups have reported that chemotherapy sensitivity can be predicted by algorithms that essentially measure levels of anti-apoptotic BCL2 family members, sum the values, and subtract levels of BAX and BAK ^105– 107^. While this approach detects differences between sensitive and resistant groups of cell lines or tumors, overlap between the groups might make it difficult to use this approach to dictate therapy for individual patients. Moreover, this approach generally fails to take into account endogenous levels of BH3-only proteins and other binding partners that could alter the anti-apoptotic or pro-apoptotic potentials of the proteins assayed.

For assessing venetoclax sensitivity of lymphoid malignancies, the calculation might actually be simpler. Myc-transformed murine lymphomas are sensitive to navitoclax only if they have elevated BCL2 ** levels ^107^. Accordingly, measurement of BCL2 alone might help predict sensitive versus resistant CLL cases. Consistent with this possibility, recent studies have reported that high BCL2 expression correlates with venetoclax sensitivity in neoplastic lymphocytes ^11, 108^. Whether elevated levels of BCLX _L_ or MCL1, either as a consequence of gene amplification ^79^ or other modifications ^109, 110^, will similarly predict sensitivity to selective antagonists of these two proteins remains to be tested.

### BH3 profiling assays

BH3 profiling involves treating mitochondria with BH3 peptides and measuring cytochrome c release or mitochondrial depolarization as a strategy to predict sensitivity to BH3 mimetics ^111, 112^ or therapies that act through inducing BH3-only proteins ^113, 114^. Because the BAD BH3 and HRK BH3 domains have different affinities for anti-apoptotic BCL2 family proteins ^44, 115^, with BAD binding BCL2 and BCLX _L_ tightly but HRK binding only BCLX _L_, subtracting the cytochrome c release caused by HRK from that caused by BAD (BAD–HRK) reportedly predicts venetoclax sensitivity ^116^. Results using this assay suggested that the maturation stage of T-ALLs determines their sensitivity to navitoclax or venetoclax, with most T-ALLs exhibiting navitoclax sensitivity but early T-cell progenitor ALL being sensitive to venetoclax ^116^. This assay also predicted that a substantial percentage of AMLs would be sensitive to venetoclax ^73^. In a subsequent phase II study of venetoclax monotherapy in AML, however, BH3 profiling results correlated only weakly with time on study ^74^, suggesting that determinants of response are more complicated than originally envisioned.

Building on the experience with BH3 profiling, a modified assay called “dynamic BH3 profiling” involves exposure of cells to diluent versus any potential anticancer drug or combination followed by assessment of mitochondrial depolarization by BIM BH3 peptide in permeabilized cells ^117^. Early experience with this assay in multiple model systems indicates that drug-induced increases in BIM BH3 peptide-induced mitochondrial depolarization after 16 hours of drug exposure correlate with the extent of cell death at 72–96 hours of continuous drug exposure *ex vivo*. Whether this assay will provide improved ability to predict response to BH3 mimetics in the clinical setting remains to be determined.

### Preformed complexes as potential predictors of response

An alternative approach to predicting BH3 mimetic sensitivity might come from recent studies demonstrating constitutive BAK activation in a variety of cells ^46, 47^. If BAK is constitutively bound to BCLX _L_, cells are significantly more sensitive to navitoclax, and if BAK is constitutively bound to MCL1, cells are more sensitive to A-1210477 ^46^, suggesting that measurement of preformed BCLX _L_·BAK and MCL1·BAK complexes might provide insight into sensitivity to the respective BH3 mimetics. There is also a correlation between preformed BCL2·BAK complexes and venetoclax sensitivity ^46^, perhaps reflecting the fact that these complexes, though somewhat less stable, nonetheless form when BCL2 is expressed at high levels or harbors gain-of-function mutations ^118, 119^. All of these complexes between BAK and anti-apoptotic BCL2 family members can, like complexes between BH3-only proteins and anti-apoptotic BCL2 family members ^44, 45^, be detected and potentially quantified by immunoprecipitation ^46^. Because anti-apoptotic BCL2 family members are expressed on the cytoplasmic surfaces of multiple organelles, not just mitochondria ^120, 121^, it is possible that immunoprecipitation followed by immunoblotting for BAK, BAX, and BH3-only proteins will provide a more complete picture of the cellular balance between pro-apoptotic and anti-apoptotic BCL2 family members than MOM permeabilization analyses alone.

## Opportunities for future development

While the recent FDA approval of venetoclax marks a milestone in apoptosis research, there is still much work to be done. As mentioned above, the results of single-agent venetoclax trials in *TP53* wild-type CLL, other lymphoid malignancies, SCLC, and ER ^+^ breast cancer are awaited with interest. Moreover, further studies examining the optimal use of venetoclax as a chemosensitizing agent are needed.

Based on the observed clinical activity of venetoclax in CLL, the prospect of selectively targeting BCLX _L_ and MCL1, especially in cancers with *BCLX* or *MCL1* amplification ^79^, is also appealing. There are, however, substantial obstacles. The role of BCLX _L_ in platelet survival and the consequent thrombocytopenia induced by BCLX _L_ inhibition hampered the development of navitoclax ^10^. Whether it will be possible to develop a clinically viable strategy for avoiding or overcoming this on-target side effect with selective BCLX _L_ inhibitors remains to be determined. Likewise, it was reported over a decade ago that MCL1 is required for hematopoietic stem cell survival ^122^. Because it now appears that this might be due to a BH3-binding groove-independent role for MCL1 in oxidative phosphorylation ^106^ rather than the role of MCL1 in apoptosis, it is possible that MCL1-selective BH3 mimetics will not be as toxic to normal cells as *MCL1* gene disruption. The development of an MCL1 inhibitor that can be applied in preclinical tumor models and possibly in clinical trials would allow this hypothesis to be tested.

Finally, a substantial fraction of tumors might be resistant to selective BCL2, BCLX _L_, or MCL1 inhibitors. To the extent that these agents act by releasing partially activated BAK or BAX from pre-formed complexes ^46^, cells lacking activated BAK and BAX will experience little or no effect from these inhibitors. One strategy for sensitizing these cells to BH3 mimetics would be to treat with chemotherapeutic agents that activate BH3-only proteins, leading to BAK or BAX activation ^46^. Alternatively, it might be possible to induce apoptosis in these cells using BH3 mimetics that directly activate BAK and/or BAX. Whether it will be possible to derive such compounds and target them in a way that allows cancer cell-selective killing also remains to be determined.
